# Perceived Healthcare Interventions and Clinical Strategies Against Dating Violence: A Qualitative Exploration of Expert and Nursing Perspectives

**DOI:** 10.3390/nursrep16090319

**Published:** 2026-09-04

**Authors:** Montserrat Puig-Llobet, Sara Sanchez-Balcells, Zaida Agüera, Maria Aurelia Sánchez-Ortega, Ana Ventosa-Ruiz, Khadija El-Abidi, Núria Vergés Bosch, Dolors Rodriguez-Martin, Nuria Rodríguez Ávila, Alba Juárez Sánchez, Elena Maestre, Mireia Perau Campos, Alba Luque, Cristina Martínez Bueno, Liliana Aragón Castro, Cristina Esteban Sanz, Marina Nebot Echeverria, Assumpta Rigol, Marta Prats-Arimon

**Affiliations:** 1 Departament de Salut Pública, Salut Mental i Maternoinfantil, Facultat d’Infermeria, Universitat de Barcelona, L’Hospitalet de Llobregat, 08907 Barcelona, Spain; monpuigllob@ub.edu (M.P.-L.); anaventosaruiz@ub.edu (A.V.-R.); kadi.elabidi@ub.edu (K.E.-A.);; 2 Research Group in Mental Health, Psychosocial and Complex Nursing Care (NURSEARCH), Facultat d’Infermeria, Universitat de Barcelona, L’Hospitalet de Llobregat, 08907 Barcelona, Spain; 3 Department of Professional Foundations and Health Management, Faculty of Health Sciences and Wellbeing, University of Vic, 08500 Vic, Spain; 4 Department of Sociology, Faculty of Economics and Business, University of Barcelona, 08034 Barcelona, Spain; nuria.verges@ub.edu (N.V.B.);; 5 Departament d’Infermeria Fonamental i Clínica, Facultat d’Infermeria, Universitat de Barcelona, 08907 Barcelona, Spain; dolorsrodriguezmart@ub.edu (D.R.-M.);; 6 Research Group in Gender, Identity and Diversity (GENI), University of Barcelona, 08907 Barcelona, Spain; 7 Psychology Services Center of the University of Barcelona, Josep Finestres Foundation, L’Hospitalet de Llobregat, 08035 Barcelona, Spain; 8 Department of Clinical and Health Psychology and Psychotherapy, Faculty of Psychology and Education Sciences, Universitat Oberta de Catalunya (UOC), 08018 Barcelona, Spain; 9 Home-Based Mental Health Care, Yomio, 08018 Barcelona, Spain; 10 Primary and Community Care Directorate, Catalan Institute of Health (ICS), 08007 Barcelona, Spain; 11 Helia Association, 08013 Barcelona, Spain; 12 Emergencies Services from Primary Care, CUAP Primary Health Care Peracamps, 08029 Barcelona, Spain; 13 Faculty of Nursing, University of Barcelona, 08907 Barcelona, Spain

**Keywords:** dating violence, intimate partner violence, nursing, early identification, professional practice

## Abstract

**Introduction**: Dating violence is an early form of intimate partner violence characterized by controlling behaviors, psychological and sexual abuse, and digital coercion. Despite its high prevalence among adolescents and young adults, it remains underrecognized and insufficiently addressed in healthcare settings. Nurses are strategically positioned to identify and respond to dating violence due to their close contact with patients and holistic approach to care. However, important gaps remain between existing protocols and their implementation in clinical practice. **Methods**: An exploratory qualitative study was conducted within a constructivist paradigm as part of a multicenter research project on dating violence among health sciences university students. Twelve experts, including nurses, healthcare professionals, and representatives of survivor-led organizations, participated in focus groups and reflective journals between September and December 2025 at the University of Barcelona. Data were analyzed using an interpretative phenomenological approach, with inductive thematic analysis guiding coding and theme development. Methodological rigor was ensured following the Consolidated Criteria for Reporting Qualitative Research (COREQ). Ethical approval was obtained. **Results**: Four themes emerged: (1) barriers to identification, including the normalization of controlling behaviors, myths of romantic love, and limited recognition of psychological and digital violence; (2) challenges in nursing responses, such as insufficient training, clinical uncertainty, organizational barriers, and lack of screening tools; (3) identification strategies, including systematic screening, relationship-centered interviewing, emotional education, and recognition of subtle warning signs; and (4) actions following identification, focused on validation, confidentiality, risk assessment, interdisciplinary referral, and ongoing support. **Conclusions**: Findings highlight the need to update protocols to explicitly address digital violence and strengthen nursing-led interventions. Participants emphasized the need for enhanced professional training, context-appropriate screening tools, and intersectoral collaboration as potential strategies to improve the identification and response to dating violence.

## 1. Introduction

Dating violence is a broad phenomenon encompassing abusive dynamics within adolescent and young adult romantic relationships. It is conceptualized as a form of intimate partner violence (IPV) that occurs specifically in dating relationships and may involve physical, psychological, economic, sexual, and digital abuse, as well as coercive and controlling behaviours perpetrated by a romantic partner. Gender-based violence represents the broader framework encompassing violence rooted in unequal gender relations, including certain manifestations of dating violence and IPV. Globally, the most recent estimates indicate that gender-based violence remains a public health issue of epidemic proportions, with nearly one in three women experiencing physical and/or sexual violence during their lifetime, a reality that affects adolescents and young women at particularly early ages [1]. In 2025, it was estimated that approximately 840 million women had experienced intimate partner violence or sexual violence, a figure that has remained largely unchanged for more than two decades, with particularly high prevalence rates in low-income regions [2]. This situation highlights the persistence of structural inequalities and the urgent need to strengthen prevention and intervention policies.

The severity of this phenomenon is also reflected in its lethal consequences. In 2023, United Nations agencies estimated that approximately 51,100 women and girls were killed by an intimate partner or family member, most of them within their own homes, equivalent to one victim every ten minutes [3]. This progression from subtle forms of psychological control to femicide underscores the continuum and cumulative nature of violence.

At the regional level, data from Catalonia reflects the same trend. Between 2012 and 2024, 156 femicides were recorded, while 9850 calls were made to the helpline for women experiencing violence between January and September 2025 alone [4]. These figures point to both a high prevalence of violence and a growing demand for support, protection, and prevention services, confirming that violence against women remains a major public health challenge.

In clinical settings, this reality becomes even more complex. Women may remain silent, deny, or struggle to recognize the violence they are experiencing, often due to fear, feelings of guilt, or the presence of the perpetrator [5]. At the same time, healthcare professionals may face uncertainty, insufficient training, a lack of protocols, or limited legal knowledge, all of which hinder early identification [6]. These barriers contribute to the invisibility of many cases of IPV within routine healthcare practice.

To address these clinical barriers, organizational frameworks such as Trauma-Informed Care (TIC) offer a strengths-based approach that assumes most service users have trauma histories, restructuring institutional policies and environments to prevent re-traumatization without requiring direct trauma processing [7,8,9]. Guided by core principles such as physical and psychological safety, transparency, peer support, collaboration, and empowerment [9,10], TIC translates into clinical practice through applied frameworks like the 4 Cs model (Calm, Contain, Care, Cope). This model shifts the fundamental clinical focus from “What is wrong with you?” to “What happened to you?” [11]. Furthermore, Trauma- and Violence-Informed Care (TVIC) extends this paradigm by explicitly accounting for the intersecting impacts of systemic violence, structural inequities, and ongoing victimization [12].

IPV has profound biological, psychological, and social consequences, ranging from acute injuries and long-term health sequelae to mental health disorders, psychosomatic symptoms, and loss of autonomy [13]. Beyond its impact on the individual, IPV also affects families and imposes substantial social and economic costs, perpetuating a cycle of harm that is difficult to break.

Given this complexity, healthcare professionals play a crucial role in early identification. Primary care and emergency services provide valuable opportunities to recognize warning signs, including psychosomatic symptoms that may reflect the chronic stress associated with exposure to violence. However, educational and organizational barriers continue to hinder a systematic and proactive approach, as documented in the literature [14].

At the same time, the nature of violence has evolved. It is now understood as a cycle of systemic control in which psychological abuse predominates, and digital technologies enable new forms of surveillance, manipulation, and harassment. This form of technology-facilitated coercive control, including cyber dating abuse, digital surveillance, and online monitoring behaviours, which often precedes physical violence, has not yet been adequately incorporated into current clinical guidelines, creating a gap between the lived experiences of victims and existing healthcare practices [1,15].

Taken together, these challenges highlight a clear disconnect between theoretical protocols and everyday clinical practice, despite the strategic position of nursing within healthcare systems. Although substantial quantitative evidence exists regarding the prevalence and mortality associated with IPV, there remains a limited understanding of the experiences, perceptions, and decision-making processes that healthcare professionals encounter in their daily practice. The barriers identified, including gaps in training, organizational constraints, and the failure to keep pace with emerging forms of control such as digital harassment, suggest that current interventions remain largely reactive and do not adequately address the contemporary complexity of dating violence. Within this context, the present qualitative study seeks to explore the perspectives of nurses and other health experts in order to identify strategies perceived as useful for improving the identification and management of dating violence.

## 2. Materials and Methods

This study employed a qualitative design aimed at exploring the perspectives of nurses and other experts in the field of intimate partner violence (IPV). The research represents the third phase of a multicenter project focused on the detection of violence within intimate relationships among university students in the health sciences. The first phase of the project, which adopted a qualitative approach, has already been published [16], while the second phase, multicenter in nature, is currently under review. The present study corresponds to the exploratory phase conducted from the perspective of nurses and other experts in the field of gender-based violence, including healthcare professionals and representatives of survivor-led organizations. The study was conducted within a constructivist paradigm and followed the methodological framework proposed by Smith and Fieldsend [17]. Within the constructivist paradigm, knowledge is understood as socially constructed through interactions, experiences, and interpretations. This perspective informed the study design by privileging participants’ subjective meanings and professional experiences regarding dating violence. Focus groups were selected to facilitate the co-construction of knowledge through group discussion, while reflective journals provided opportunities for individual reflexivity and meaning-making. Consistent with this paradigm, data analysis focused on interpreting how participants understood and negotiated their professional experiences rather than identifying a single objective reality. The researchers adopted a reflexive stance throughout the analytical process, acknowledging their active role in the co-construction and interpretation of findings. This study is reported in accordance with the Consolidated Criteria for Reporting Qualitative Research (COREQ), ensuring transparency, comprehensiveness, and methodological rigor in the presentation of the findings [18].

The project received approval from the Bioethics Committee of the University of Barcelona (Institutional Review Board IRB00003099), under reference code CER0624-07. All potentially identifying information was anonymized to protect participant privacy. Participation was voluntary, and all participants provided written informed consent prior to taking part in the study.

### 2.1. Study Setting and Recruitment

The study was conducted at the University of Barcelona (Barcelona, Spain). Prior to its implementation, an informational meeting was held with all participating nurses and other experts in the field, during which the objectives of the project were presented, and explanatory documents related to the study were provided.

Participants were recruited exclusively on the basis of their status as practicing nurses or as experts with current or previous experience providing care to young people, rather than on any personal experiences related to dating violence. Recruitment and the focus groups were conducted outside participants’ working hours to ensure voluntary participation and to avoid any interference with their clinical responsibilities.

Participants were recruited from a range of healthcare institutions, leading community organizations, and nursing schools across the Barcelona metropolitan area. A purposive sampling strategy was employed to recruit professionals with demonstrated expertise in intimate partner violence, gender-based violence, mental health, community care, education, or victim support services. Several members of the research team from the same geographical region coordinated the sessions, which were moderated in both Catalan and Spanish. The focus groups were conducted in a private setting to ensure confidentiality, safeguard the well-being of the nurses and other health experts, and prevent the presence of external individuals who might influence participants’ responses.

A total of 12 professionals from nursing and other disciplines were selected to participate in the study. Initial contact was established through management teams at healthcare institutions and professional networks, which facilitated communication with the research team. The inclusion of multidisciplinary experts alongside nurses was intentional, as dating violence is a complex phenomenon that requires coordinated responses across healthcare, social, educational, and community settings. This diversity of professional perspectives enabled a more comprehensive exploration of barriers, facilitators, and opportunities for intervention. Sample size was guided by the principle of information power, as participants possessed substantial experience and expertise directly related to the study aims, allowing for the collection of rich and relevant data. All participants confirmed their willingness to take part by signing an informed consent form. Two initially identified candidates were excluded because they did not complete this process, and one was unable to attend the scheduled session due to clinical work commitments.

Participants ranged in age from 33 to 72 years. All participants identified as women. In terms of professional background, the group included nurses, psychologists, technical specialists, and university faculty members with diverse experience across clinical, community, academic, and nonprofit settings. Participants were employed in a variety of professional contexts, including healthcare services (emergency care, mental health, and community care), higher education, social organizations, and private practice, providing a broad and complementary range of perspectives that enriched the analysis. Table 1 presents the detailed characteristics of the participants.

### 2.2. Data Collection

Focus groups were selected as the primary method of data collection. This methodological choice was based on the value of collective discourse over individual accounts, as group interaction fosters shared reflection and the co-construction of knowledge.

The focus groups were facilitated by the principal investigator (MP-L), a faculty member holding a doctoral degree with expertise in research on egalitarian relationships and extensive experience conducting focus groups. A second researcher (SSB), who holds a PhD from the University of Barcelona and whose research focuses on intimate partner violence (IPV), participated as an observer. Together, these researchers were responsible for planning and conducting the sessions between September and December 2025. Prior to the study, some researchers and participants already knew one another through their shared involvement in the university’s Equality Committee. No participant was involved in the research team, and no researcher was included as a participant in the study. To minimise the potential influence of these pre-existing professional relationships, participants were informed that all contributions would remain confidential and would be anonymised during analysis. The use of a semi-structured focus group guide ensured consistency across discussions, while reflective journals provided an additional opportunity for participants to express their perspectives independently and in greater depth. The meetings were held in a private room at the Faculty of Nursing, ensuring appropriate conditions of comfort and confidentiality. Each focus group session lasted approximately 90 to 120 min. A thematic guide (Supplementary File S1) was developed using questions derived from the literature on dating violence [19,20,21].

The focus groups were audio-recorded and subsequently transcribed verbatim by the principal investigator (MP-L) using transcription software TurboScribe (https://turboscribe.ai, web-based AI transcription software, accessed on 31 December 2025), followed by a thorough manual review to ensure accuracy. At the beginning of each session, participants were informed of the objectives of the group and were provided with a document outlining the topics to be addressed. The audio recordings and reflective journals were transcribed in full by the principal investigator using a digital recorder. To protect confidentiality and anonymity, each participant was assigned a pseudonym during the transcription and analysis process.

Following completion of the focus groups, participating experts were invited to complete a reflective journal as a second method of data collection. This activity involved a process of in-depth personal reflection during the data collection period, as participants worked directly with women experiencing gender-based violence in their everyday professional practice and regularly encountered situations requiring detection, support, and intervention. As such, the reflective journal served as an essential tool for understanding how participants interpreted and developed strategies and approaches for addressing gender-based violence based on their lived professional experiences.

Two focus groups were conducted involving a total of 12 participants. All participants were invited to complete a reflective journal, and seven participants submitted at least one journal entry. Reflective journals were approximately 1500 words in length and provided complementary insights into participants’ professional experiences. Although the reflective journals invited participants to reflect on their broader professional experiences of gender-based violence, only excerpts relevant to intimate partner violence and associated processes of identification, prevention, and intervention were included in the analysis. Broader reflections were retained only when participants explicitly connected them to the experiences and needs of adolescents and young adults in dating relationships. To support this process, the research team developed a reflective journal template that participants could complete throughout the study, documenting their interactions with women affected by gender-based violence (Supplementary File S2). The purpose of the journal was to enable health experts to engage in critical reflection on their approaches to clinical practice and intervention [22]. Reflective journals contained reflections on professional experiences rather than patient case reports. Participants were explicitly instructed not to include identifiable information, and all materials were anonymized before analysis.

Upon completion, the reflective journals were submitted by email to the principal investigator (MP-L) and incorporated into the qualitative analysis alongside the focus group transcripts, following the same coding and categorization procedures. Data collection took place between September and December 2025.

Data collection and analysis were conducted iteratively. Information power was considered sufficient when recurring patterns were consistently identified across focus groups, reflective journals, and field notes, and no substantially new insights relevant to the study objectives emerged.

### 2.3. Data Analysis

The study was informed by a constructivist and interpretative phenomenological perspective, while data were analysed using reflexive thematic analysis following Braun and Clarke [23,24]. The research team engaged in iterative coding, reflexive discussions, and theme refinement to interpret the meanings participants attributed to dating violence identification and intervention.

Focus group transcripts and reflective journals were analyzed together using the same coding framework. Codes derived from one data source were continuously compared with those from the other source to identify convergences and divergences across participants’ accounts. Reflective journals largely confirmed and enriched themes emerging from the focus groups by providing more detailed individual reflections. In some cases, the journals contributed additional nuances and contextual insights that helped refine existing themes. Any divergences identified between data sources were explored through reflexive team discussions and interpreted as complementary perspectives rather than inconsistencies.

The analytical process was structured into four phases. In the first phase, all transcripts and reflective journals were read repeatedly and in depth to facilitate familiarisation with the data. Materials were stored and managed using Atlas.ti^®^ 26.1.0 qualitative data analysis software (Atlas.ti Scientific Software Development GmbH, Berlin, Germany) [25]. In the second phase, relevant excerpts were coded using an inductive approach guided by the research questions on dating violence. During this stage, themes related to digital violence, less visible forms of dating violence, and the consequences of abuse for psychological well-being emerged from participants’ accounts.

Subsequently, the coded excerpts were reviewed inductively to identify themes and subthemes that captured key concepts, as well as similarities and differences in the attitudes and perspectives expressed by participants. Third, codes were reviewed and organised into preliminary themes and subthemes that captured key concepts, as well as similarities and differences in participants’ perspectives. Finally, themes and subthemes were refined, defined, and named to accurately represent participants’ understandings of the barriers, challenges, and strategies associated with the identification and management of dating violence. Selected quotations were edited minimally to improve readability while preserving their original meaning.

The research team conducted the coding and theme development process collaboratively and was coordinated by the principal investigator (MP-L). Team members had previous experience in mental health nursing, qualitative research, and gender-based violence, which informed their sensitivity to the topic while requiring ongoing reflexive awareness of potential assumptions and preconceptions. Regular analytical meetings were held to discuss coding decisions, compare interpretations, and critically examine emerging themes. Differences in coding or interpretation were discussed until consensus was reached. These reflexive discussions contributed to refining thematic boundaries, challenging preliminary assumptions, and ensuring that the final interpretation remained grounded in participants’ accounts. Throughout the entire process, qualitative data analysis software (Atlas.ti^®^) was used to support data management and analysis. All data were collected and analyzed in Catalan and Spanish. Quotations included in the manuscript were translated into English by the research team and reviewed by bilingual researchers to ensure conceptual equivalence.

Throughout the analytical process, the research team continuously reviewed emerging codes, themes, and patterns across focus group transcripts and reflective journals. Data were considered sufficient to address the study aims when additional analysis no longer generated substantially new themes or perspectives relevant to the identification and management of dating violence. This decision was reached collectively through ongoing discussions among the research team and informed by the recurrence and richness of concepts identified across the dataset.

### 2.4. Rigor and Reflexivity

The principal investigator’s background in research on egalitarian relationships and interventions with mental health populations shaped the theoretical approach adopted toward dating violence. This perspective informed both the development of the focus group guides and the interpretation of participants’ narratives. The second researcher, a PhD-trained specialist in mental health, guided the process toward a collaborative epistemological approach to knowledge construction. The research team acknowledges that these perspectives may have influenced the interpretation of the data. However, to minimize potential biases associated with the researchers’ backgrounds, dedicated spaces for explicit discussion were incorporated into team meetings, reflective field journals were maintained throughout the study, and ongoing critical scrutiny was applied during the analytical process.

Some members of the research team had prior professional relationships with several participants through their shared involvement in the University’s Equality Committee. To minimise the potential influence of these relationships, participants were informed that all contributions would remain confidential and would be anonymised during analysis. During data collection, a semi-structured focus group guide was used to ensure consistency across discussions, while reflective journals provided participants with an additional opportunity to express their perspectives independently and in greater depth. During analysis, investigator triangulation, reflexive discussions, and collaborative theme development were used to critically examine emerging interpretations and minimise the influence of pre-existing assumptions or relationships on the findings.

Several strategies were employed to ensure methodological rigor. First, investigator triangulation was used: the principal author (MP-L) led the coding process and the development of the thematic framework, both of which underwent extensive review and discussion with the co-investigators until the research team reached agreement that no substantially new themes were emerging from the data. Second, member checking was conducted by presenting preliminary findings to participants to validate the interpretations and strengthen the credibility of the study. Finally, auditability was ensured through a detailed record of the decision-making process, comprehensively documented in fieldwork journals and reflective notes.

In addition, the study adhered to the standards established by the Consolidated Criteria for Reporting Qualitative Research (COREQ) checklist, which is available in the Supplementary Materials.

## 3. Results

The findings derived from the qualitative analysis are presented below. Table 2 summarizes the main themes and their conceptual definitions, providing an overview of the analytical structure underpinning the findings. To enhance transparency and rigor in the reporting of the analytical process, Table 3 illustrates the progression from initial codes to categories, themes, and the final conceptual framework generated from the data.

### 3.1. Barriers to Identifying Dating Violence

The narratives reveal that the invisibility of violence is not an isolated phenomenon but rather the result of a complex network of barriers operating at the individual, relational, social, and professional levels. Most participants described normalization and invisibility as major barriers to identifying dating violence. First, psychological and perceptual barriers emerged, characterized by the normalization and minimization of controlling behaviors that are interpreted as expressions of care or affection. These perceptions are reinforced by myths of romantic love that sustain the belief that possessiveness is a sign of love and that love can overcome any obstacle. This perspective makes it particularly difficult to recognize nonphysical forms of violence, as many young women identify abuse only when explicit aggression occurs. At the same time, the delegitimization of their own experiences, rooted in gender socialization, leads them to question their perceptions and feel responsible for their distress. As participant P5 explained: “*one of the main barriers is the invisibilities and normalization of violence in young people’s relationships, especially when it is presented as jealousy or ‘affectionate’ control.*” (P5, Focus group).

At the social and gender levels, the persistence of patriarchal norms and binary constructions of sex continue to shape behaviors that perpetuate inequality, while digital environments and pornography facilitate virtual forms of control and normalize nonconsensual practices that become embedded as accepted relationship standards. These dynamics are further reinforced by peer pressure and what participants described as “social noise,” whereby fear of rejection, exposure on social media, and substance use in leisure settings obscure warning signs and make abusive behaviors more difficult to recognize.

As participant P2 noted: “*Social noise refers to environmental pressure, the normalization of violence in nightlife settings, and alcohol and drug use, all of which blur the warning signs.*” (P2, Reflective Journal).

Important barriers also emerged within professional and clinical settings. These included diagnostic uncertainty, which can lead clinicians to attribute distress to mental health disorders when its underlying cause is actually exposure to violence; structural constraints such as limited time, insufficient supervision, and a lack of practical tools; and professional reluctance stemming from concerns about addressing sensitive topics or uncertainty regarding how to manage them appropriately. As participant P1 explained: “*In the professional setting, there is a lack of time and supervision opportunities, as well as a lingering reluctance to address a topic that may generate resistance*.” (P1, Focus Group).

Finally, participants working in educational and academic settings particularly emphasized the role of consent education and the need to challenge myths of romantic love. This makes it more difficult for them to recognize healthy boundaries and to fully exercise self-determination in relation to leisure, relationships, and sexual desire. In this context, one nurse emphasized: *“Explicit consent must be placed at the center of relationships and respect for boundaries.”* (P6, Focus Group).

### 3.2. Challenges in Addressing Intimate Partner Violence (IPV)

The challenges involved in addressing dating violence among young people constitute a complex web of individual, relational, institutional, and structural factors. Nurses and other experts emphasized the importance of empathy and the avoidance of judgment by institutions and professionals, as the generational gap often leads young people to perceive adults as figures who question rather than support them. This perception reduces trust and reinforces the belief that institutions may represent an obstacle rather than a source of help. As one participant stated: “*The lack of people capable of listening respectfully, empathetically, and without judgment can seriously hinder both the identification of violence and the ability to leave abusive situations.*” (P3, Reflective Journal).

These challenges are compounded by the normalization of controlling and possessive behaviors, often reinforced through social media, as well as by a lack of awareness regarding healthy boundaries shaped by toxic relationship models. In professional practice, resistance to discussing the issue is common due to shame, emotional dependence, or fear of being judged. As a result, many women retrospectively recognize early signs of abuse that they failed to identify during adolescence or young adulthood. These difficulties are also reflected in feelings of helplessness, self-blame, fear of not being believed, social isolation, and emotional exhaustion, all of which are exacerbated by the absence of supportive networks. As one participant noted: “*The absence or weakness of support networks means that many young women lack an environment that conveys the message, ‘I believe you.’*” (P3, Focus Group).

At the societal level, additional challenges include the denial or minimization of the problem by victims themselves or by their peers, the cultural normalization of psychological violence, particularly within digital environments, the perception that adults “will not understand,” and the difficulty of creating safe spaces in which to discuss consent and sexuality. At the same time, fear of social consequences often reinforces silence and discourages disclosure. One participant described this situation as follows: “*Young people may be deeply immersed in digital environments that facilitate invisible forms of control. At present, we do not have specific tools to detect these dynamics in digital settings, nor are screening protocols adapted to this environment being implemented.*” (P11, Focus Group).

At the institutional level, participants highlighted the limited implementation of educational protocols due to a lack of specialized training, poor coordination among social, healthcare, and educational services, shortages of human and material resources, insufficient time and appropriate spaces, and gaps in the emotional education of both young people and adults. Across professional backgrounds, participants consistently reported inadequate training, limited institutional support, and poor intersectoral coordination as significant challenges for addressing dating violence. These shortcomings often contribute to paternalistic or otherwise inadequate responses. One participant described the situation as follows: “*There is a clear lack of emotional education: adolescent girls and young women are not prepared to recognize abusive behaviors, and the adults around them often respond without appropriate strategies or with overly paternalistic attitudes.*” (P10, Focus Group).

Intervention efforts are further complicated by subtle and often invisible forms of sexism, stigma, fear of rejection, deep engagement with digital environments where covert forms of control are difficult to detect, and the lack of tools specifically designed for digital screening. These challenges are compounded by emotional strain and professional burnout, resistance from both victims and their social networks, mistrust of protection services, concerns about confidentiality, and resistance to change on the part of both the victim and the perpetrator. As a result, interventions may shift toward attempts to “improve the relationship” rather than addressing gender-based violence itself. One participant summarized this issue as follows: *“Both the perpetrator and the victim may deny or minimize the abuse, which can divert interventions away from the underlying dynamics of power and control.”* (P5, Reflective Journal).

### 3.3. Potential Strategies for Identifying Dating Violence

Based on participants’ narratives, the strategies identified for detecting dating violence among young people are organized around a comprehensive approach that prioritizes primary prevention, the strengthening of socioemotional competencies, and the optimization of institutional mechanisms. Participants’ narratives and reflections emphasized that effective identification depends on the implementation of systematic screening protocols in educational and healthcare settings, using clinical interviews based on open-ended, nonintrusive questions that facilitate discussion of relationship quality without causing revictimization. Particular importance was placed on training professionals to recognize subtle warning signs, such as social isolation, anxiety, and mood changes, as well as to effectively manage rapid referral pathways among primary care, mental health services, and social support systems. As one participant noted: *“Professionals should be trained to ask open, non-invasive questions about young people’s relationships and strengthen coordination across services.”* (P2, Reflective Journal).

A key finding was the need to address digital violence and social media pressure, phenomena that require the analysis of real-life situations involving online control as well as the dissemination of awareness campaigns focused on less visible forms of abuse. Several participants highlighted the need for screening and assessment tools adapted to digital environments, reflecting growing concerns about technology-facilitated forms of abuse. One participant highlighted: *“Digital environments facilitate invisible forms of control, yet we still lack screening and assessment tools that are adapted to these realities. Young people are highly connected to social media, and detection strategies must also evolve to address these spaces.”* (P6, Reflective Journal).

Participants’ accounts also suggest that group-based interventions and emotional education are critical tools for challenging myths of romantic love and the patriarchal stereotypes that underpin violence. As one participant reflected: *“Creating spaces of trust and fostering narratives that reduce self-blame help young people put a name to their experiences of abuse, overcome isolation, and strengthen their ability to protect themselves from both digital and physical forms of harassment.”* (P2, Focus Group).

Another participant emphasized: “*Educational interventions should challenge myths of romantic love, promote explicit consent, and strengthen young people’s autonomy and ability to establish healthy boundaries within relationships.”* (P3, Reflective Journal).

Finally, participants proposed that educational institutions should function as safe environments through the ongoing training of educators and the provision of accessible and confidential information points. This approach includes the integration of updated educational materials that promote a gender-sensitive perspective, clearly define the various manifestations of violence to prevent their trivialization, and support the availability of psychological services that operate without bureaucratic or parental barriers, thereby ensuring an immediate response in situations involving risk.

### 3.4. Actions Following the Identification of Intimate Partner Violence (IPV)

Actions following the identification of dating violence require a coordinated, sensitive, and youth-centered approach in which educational institutions, social services, and healthcare settings ensure that their protocols function as effective tools adapted to the needs of young people. As one participant stated, “*Educational institutions and social services must be capable of ensuring that their protocols work and of adapting them when necessary; providing appropriate support is essential.*” (P4, Reflective Journal).

Nearly all participants emphasized the importance of validation, confidentiality, and multidisciplinary collaboration following the identification of dating violence. Participants’ narratives emphasized the importance of immediately validating the experiences of those affected, avoiding revictimization, and providing clear and accessible information about available resources. One professional explained: “*When I identify a possible situation of violence, I consider it essential to validate the person’s experience, avoid revictimization, and provide clear, tailored information.*” (P1, Focus Group). This process should always ensure confidentiality and respectful emotional support. Another participant added: “*It is necessary to create spaces for listening, validate without judgment, and respect the individual’s pace; they should not be pressured to end the relationship.*” (P9, Reflective Journal). Professionals also emphasized the need to assess risk carefully, activate rapid referral pathways, and coordinate with multidisciplinary teams. As one participant explained: “*I usually coordinate with the multidisciplinary team and activate referral pathways when necessary to ensure safety and continuity of care.*” (P12, Focus Group).

Within the field of mental health, it is essential to assess how experiences of violence may influence the course of a disorder, treatment adherence, and self-concept. As one professional summarized: “*In mental health settings, it is particularly important to assess how violence may have affected the disorder or adherence to treatment.*” (P3, Reflective Journal). Participants with mental health expertise particularly highlighted the risk of misattributing violence-related distress to psychiatric symptoms. In this context, participants also highlighted the importance of providing emotional support to professionals themselves, as ongoing exposure to cases of violence can lead to significant emotional strain and burnout. As one participant noted: “*Professional supervision and emotional support are necessary because working with these cases has an emotional impact.*” (P2, Reflective Journal). Participants’ narratives also underscored the importance of creating spaces for listening, facilitating support networks, and accompanying individuals throughout what are often lengthy processes of recovery and change. As one participant stated: “*Support the person while understanding that the process may be long, facilitate access to support networks, and provide information about available resources.*” (P6, Reflective Journal). Within educational and university settings, participants considered it essential to integrate a gender perspective across the curriculum and to implement workshops, role-playing activities, and other educational initiatives that help students understand the dynamics of violence, recognize warning signs, and develop strategies for self-protection. As one expert explained: “*It is necessary to incorporate a gender perspective into all subjects and introduce workshops and role-playing activities to enhance understanding of violence.*” (P8, Focus Group).

Participants also emphasized the importance of increasing the visibility of resources such as Purple Points “Punts Lila” and other safe support spaces: “*These spaces must have clear protocols, be visible and accessible, and provide immediate and safe support.*” (P2, Reflective Journal). Professionals working in community and support services particularly stressed the importance of accessible referral resources and safe support spaces. Regarding the digital dimension, participants stressed that many forms of violence are now manifested through social media and virtual environments: “*All forms of violence can have a digital expression, and young people are particularly vulnerable to these forms of abuse.*” (P12, Focus Group). Work with families, training on alternative and healthy models of masculinity, and the co-creation of programs with young people were identified as key strategies for transforming the patriarchal norms that sustain violence. As one participant reflected: “*Prevention must include the deconstruction of hegemonic masculinities and engage boys and young men as active agents of change.*” (P7, Reflective Journal).

Finally, participants emphasized that responses to dating violence cannot be limited to the management of individual cases but must address the issue as a structural, community, and systemic problem. As one participant stated: “*Dating violence is part of a broader structural problem; only a community-based approach can transform the norms that sustain it.*” (P5, Focus Group).

## 4. Discussion

The primary aim of this qualitative study was to deepen understanding of nurses’ and other health experts’ perspectives regarding strategies that may facilitate the identification and management of dating violence and support earlier intervention. Participants suggested that dating violence among young people is not a superficial phenomenon but rather remains largely hidden due to the complex interaction of psychological, social, digital, and structural factors. This complexity is consistent with the work of Santos et al. (2018) [26], who argue that failures in detection do not arise from a single cause but from a “network of resistances” that complicates healthcare responses and intervention.

Our findings highlight that psychological factors do not operate in isolation; rather, they generate an emotional impact that contributes to the construction of a “wall of silence.” As noted by Grigaitė [27], this silence is bidirectional: individuals experiencing abuse may fail to recognize it because of its emotional burden, while healthcare professionals may refrain from asking about it due to the absence of a safe framework for discussion. In this regard, the normalization of controlling behaviors, sustained by myths of romantic love, emerges as the most persistent barrier. The results of this study underscore that the belief that possessiveness is a form of affection remains deeply embedded within the university context, leading abusive behaviors to be reinterpreted as expressions of love, care, or excessive concern [27,28,29].

A noteworthy finding, consistent with the work of Briones-Vozmediano et al. [29], is the characterization of so-called “affectionate control” as an early and subtle manifestation of abuse. It is precisely this gradual progression that facilitates its acceptance and makes it more difficult to identify as a clinical concern. When controlling behaviors appear in fragmented forms, such as monitoring schedules, criticizing clothing choices, or supervising friendships, young women are less likely to perceive them as violence and more likely to interpret them as relational dynamics associated with the intensity of a romantic relationship. Likewise, framing violence as a “private matter” shifts its management outside the public and institutional spheres. The experts in our study agreed that this normalization contributes to the minimization of harm [29]. At the same time, myths associated with traditional gender roles reinforce feelings of guilt and self-doubt. Women continue to experience pressure to assume responsibility for maintaining harmony within the relationship, leading them to question the legitimacy of their own distress [29]. As highlighted in the literature [27], prolonged exposure to these normalized forms of control generates microtraumas that alter perceptions of reality and foster a growing tolerance for suffering. In the present study, this phenomenon emerged as a major barrier to autonomous help-seeking and support utilization.

Within the context of the digital era, the narratives analyzed indicate that the online environment is not a separate sphere but rather an extension of offline patterns of control. Continuous engagement with social media facilitates persistent forms of surveillance, such as real-time location tracking and the monitoring of interactions on public profiles, which are extremely difficult to detect during routine clinical encounters. These findings are aligned with emerging evidence on technology-facilitated coercive control [30], including cyber dating abuse [31], digital surveillance [32], and online monitoring behaviours [33], which are increasingly recognized as significant forms of abuse within young people’s intimate relationships. Despite growing awareness of digital violence, its identification remains challenging because many of these behaviours are normalized within contemporary digital cultures and are frequently perceived as signs of care, trust, or relationship involvement rather than indicators of abuse [34]. Furthermore, the rapid evolution of digital platforms often outpaces the development of clinical screening tools and professional training, limiting healthcare professionals’ ability to recognize and address these forms of coercive control during routine assessments [35].

The literature suggests that digital media contribute to the cultural legitimization of these behaviors by reinforcing stereotypes and promoting desensitization to violence. However, in contrast to this predominantly negative perspective, both the scientific evidence [36] and our findings indicate that digital environments also hold transformative potential. Proposed strategies include the integration of screening tools into electronic health records and the use of applications that allow young people to anonymously assess the quality of their relationships [37]. According to the nurses and other experts consulted in this study, the key is not to prohibit technology but rather to foster digital literacy among both professionals and users.

One of the most critical issues emerging from this discussion concerns the challenges associated with professional intervention. Significant structural barriers continue to contribute to the invisibility of dating violence within healthcare systems [29]. Despite its high prevalence, there remains a gap in academic and professional training that generates a sense of “technical insecurity” among practitioners [29,38,39], who frequently report concerns about legal consequences and uncertainty regarding how to manage emotional distress during disclosure. Adopting a Trauma-Informed Care (TIC) framework directly mitigates this uncertainty by prioritizing psychological safety over coercive questioning [7,10]. Specifically, operationalizing the 4 Cs model (Calm, Contain, Care, Cope) offers practitioners a clear, structured roadmap to navigate emotional disclosures without fear of re-traumatization [11], while a TVIC lens helps reframe these encounters within broader structural realities [12].

Within the field of mental health, both the literature and our findings point to a concerning tendency toward a predominantly biomedical approach [36]. Manifestations of violence, such as anxiety, insomnia, and somatic symptoms, are often treated as isolated pathologies rather than as consequences of abusive relationships [27]. As a result, emotional distress becomes overmedicalized, with treatment directed at symptoms while the underlying relational causes remain unaddressed [27]. As noted by Otero-García et al. [40], this decontextualization of suffering is not only ineffective but may also be iatrogenic, as it obscures the true source of trauma [29]. Professionals’ reluctance to initiate conversations that they feel ill-equipped to manage further widens the gap between the potential for detection and the delivery of effective intervention [29].

To overcome these barriers, the findings of the present study support a paradigm shift toward more participatory approaches [36]. The generational gap emerged as a significant obstacle to building trust; consequently, strategies such as edutainment (education through entertainment) [36], role-playing activities, and the use of standardized patients in simulation-based learning have been identified as effective resources [41]. These tools help reduce generational distance and enable young people to engage emotionally with the content without feeling judged [38]. Furthermore, they contribute to the development of key nursing competencies related to violence prevention, early identification, empathic communication, risk assessment, and referral, which are highlighted in international recommendations for professional education and training [42,43,44].

The creation of support and reflection groups within healthcare settings represents another key strategy. These spaces make it possible to work with women who do not self-identify as victims, fostering validation of their experiences through shared reflection and peer support [36]. Likewise, engaging men in prevention efforts is essential for the deconstruction of hegemonic masculinities. As highlighted by participants, prevention cannot be truly effective unless it actively involves boys and men as agents of change, challenging the notion that gender based violence is solely “a women’s issue”.

Regarding actions following the identification of violence, the findings are consistent with the recommendations of Grigaitė et al. [27] and Li et al. [35], emphasizing that immediate validation is a cornerstone of nursing practice. Healthcare professionals should act as supportive figures, avoiding pressure on victims to end the relationship immediately, as such pressure is often counterproductive and may increase the likelihood of disengagement from follow-up care and support services [36].

The findings also underscore the urgent need for protocols that promote a multidisciplinary approach and ensure conditions of complete privacy. In the absence of clearly defined care pathways, continuity of care is often left to the individual initiative of healthcare professionals, resulting in fragmented and inconsistent service provision [39].

The difficulties reported by participants in implementing existing protocols may be understood through implementation science perspectives. Frameworks such as the Consolidated Framework for Implementation Research (CFIR) [45], the Knowledge-to-Action Framework [46], and Normalization Process Theory [47] suggest that successful implementation depends not only on the availability of protocols but also on factors such as training, organizational support, resource availability, professional engagement, and integration into routine clinical workflows. This may partly explain the persistent gap between policy recommendations and everyday practice identified in the present study. The findings also suggest that organizational culture plays a critical role in shaping responses to dating violence. While protocols provide important guidance, participants described barriers related to limited institutional support, insufficient supervision, competing clinical demands, and uncertainty regarding professional responsibilities. These findings highlight that leadership commitment, supportive workplace climates, and organizational readiness may be as important as protocol availability in achieving sustainable changes in practice [48].

Coordination among social, educational, and healthcare services therefore becomes the cornerstone of ensuring that individuals do not become lost within the system [36].

This study has several limitations that should be considered when interpreting its findings. First, due to its qualitative nature and the use of purposive sampling, the aim was not statistical generalization but rather an in-depth understanding of professional experiences and perspectives. Furthermore, a degree of selection bias may have been present, as participants who agreed to take part in the study may have had a particular interest in gender-based violence and related issues, potentially resulting in more favorable attitudes and greater awareness than those typically found among healthcare professionals. Nevertheless, the sample consisted predominantly of women with a strong awareness of gender-related issues, which may have encouraged narratives aligned with feminist perspectives while limiting the emergence of divergent viewpoints or expressions of professional resistance. In addition, the sociocultural context in which the study was conducted may affect the transferability of the findings to regions with different regulatory frameworks or resource availability. Another limitation relates to the rapid evolution of digital violence, whose dynamics change so quickly that they may only have been captured partially within the scope of this research. Finally, because the study focused exclusively on professional perspectives, it does not include the direct voices of young people themselves. Consequently, the analysis of their processes of recognizing abuse and seeking help is necessarily mediated through an adult professional lens.

These limitations point to several important avenues for future research on dating violence among young people. First, it is essential to incorporate the voices of young people themselves through qualitative or mixed-methods studies that employ participatory action research approaches, enabling a deeper understanding of the forms of resistance and silence that emerge within their relational contexts. Further research is also needed on emerging forms of digital violence and their intersection with factors such as LGBTQ+ identities, migration status, and disability, alongside investigations into adolescent masculinities and the role of boys and young men as agents of prevention. At the institutional level, evaluative studies are required to assess the real-world impact of existing protocols and to examine the emotional consequences of this work for professionals. Finally, future efforts should focus on the co-creation of interventions with young people themselves, evaluating the effectiveness of affective-sexual and digital education programs in promoting relationships grounded in consent, equality, and mutual respect.

## 5. Conclusions

The analysis of the narratives provided by nurses and other experts in the fields of health and social sciences enabled the identification of key dimensions related to dating violence and IPV. Drawing on their clinical and professional experience, participants described significant barriers to the identification of violence, as well as challenges associated with its management once detected. Participants also highlighted a range of strategies that may support earlier identification and guide professional responses following detection. Taken together, these perspectives illustrate the complexity of the phenomenon and highlight the potential value of comprehensive approaches to detection, prevention, and intervention within healthcare settings.

Participants’ narratives suggested that responses to dating violence may benefit from moving beyond an exclusively individual clinical approach towards a more systemic framework centred on validation, autonomy, and interinstitutional coordination. The findings suggest that dating violence should be considered a routine component of assessment and care rather than an exceptional circumstance, particularly given the frequent normalization and invisibility of abuse within young people’s relationships. Participants also emphasized that effective identification depends not only on individual professional competencies but also on organizational readiness, including institutional support, clear referral pathways, adequate training, and multidisciplinary collaboration.

The study further highlights the growing relevance of digital coercive control as an emerging form of abuse that challenges traditional approaches to identification and intervention. According to participants, healthcare professionals require ongoing education and updated tools to recognize and respond to technology-facilitated forms of violence. Finally, participants emphasized the importance of integrating digital literacy, comprehensive affective and sexual education, the empowerment of young women, and the active involvement of boys and men in challenging hegemonic masculinities. Sustainable improvements in prevention and care are likely to require coordinated efforts across education, healthcare, community services, institutional leadership, and public policy.

Future initiatives should actively involve young people in the co-design of prevention, detection, and support strategies to ensure their relevance to contemporary social and digital environments.

### Implications for Clinical Practice

At the clinical level, participants’ perspectives underscore the potential value of a transdisciplinary, gender-transformative approach to professional practice that prioritizes the early identification of subtle forms of violence and technology-facilitated coercive control, which are often normalized through myths of romantic love. Participants emphasized the importance of strengthening diagnostic and clinical competencies through ongoing professional training that may help practitioners distinguish relational distress from psychopathology while creating safe environments that respect young women’s autonomy and readiness for change without causing revictimization. These findings support the development of competencies for nurses addressing dating violence, including skills related to trauma-informed communication, confidential interviewing, risk assessment, validation, referral, digital violence identification, and interdisciplinary collaboration. At the organizational level, participants highlighted the importance of coordinated referral pathways, institutional support mechanisms, and professional supervision structures aimed at reducing emotional burden and preventing burnout among practitioners. Furthermore, the integration of digital violence assessment into electronic health records and the development of validated digital screening tools may facilitate earlier identification and more systematic intervention. Participants additionally emphasized the value of interprofessional education and training involving nursing, psychology, medicine, and social work to strengthen coordinated responses across sectors. At the educational and community level, participants suggested that participatory affective and sexual education, routine simulation-based learning opportunities, and initiatives that challenge hegemonic masculinities and address contemporary digital forms of abuse may represent valuable components of prevention efforts in settings where young people interact. They also highlighted the importance of promoting digital literacy, consent education, healthy relationship models, peer support initiatives, and the active involvement of boys and young men in violence prevention. At the policy level, participants’ narratives suggest that sustainable improvements require coordinated action across healthcare, educational, social, and community systems. Strengthening institutional leadership, intersectoral collaboration, governance mechanisms, and adequate resource allocation may support the implementation and sustainability of prevention, identification, referral, and intervention strategies. Future research should include patient and public involvement in intervention design and evaluate the long-term implementation and effectiveness of prevention and intervention strategies across healthcare, educational, and community settings.

## Figures and Tables

**Table 1:** Professional Characteristics of Focus Group Participants.

Participant Number	Professional Category	Workplace	Years in Profession
1	University Professor	Faculty of Economics and Business	25
2	Mental Health Nurse	Mental Health Home Hospitalization Unit	15
3	Technical Specialist	Hèlia Association	25
4	Honorary Professor, University of Barcelona (Mental Health Nurse)	Retired	38
5	Nurse	Peracamps Emergency Primary Care Center (CUAP Peracamps)	21
6	Midwife and Educator	Director of ASSIR Catalonia (Catalan Health Institute) and Professor, Faculty of Nursing, University of Barcelona	39
7	Psychologist	Josep Finestres Foundation	3
8	Clinical and Forensic Psychologist	Open University of Catalonia (UOC), Women’s Information and Support Service (SIAD), Private Practice	22
9	Adjunct Professor	Faculty of Nursing	25
10	University Professor	Faculty of Nursing	25
11	Professor	Faculty of Health Sciences and Well-being	35
12	Nurse	Emergency and Sports Medicine Unit	24

**Table 2:** Themes and Conceptual Definitions.

Themes	Definition
1. Barriers to Identifying Dating Violence	Individual, social, and structural factors that hinder the recognition of abuse. This theme includes the normalization of controlling behaviors through myths of romantic love, the invisibility of nonphysical forms of violence, and “social noise” (peer pressure and the normalization of such behaviors within social and leisure contexts).
2. Challenges in Addressing Intimate Partner Violence (IPV)	Barriers to professional intervention, including a lack of specialized training, an overly biomedical approach to mental health care, the generational gap between professionals and young people, and concerns about case follow-up due to insufficient supervision and support.
3. Potential Strategies for Identifying Dating Violence	Preventive and diagnostic actions that prioritize emotional education and systematic screening. This theme highlights the use of digital tools, the deconstruction of hegemonic masculinities, and the use of edutainment resources to reduce the generational gap between professionals and young people.
4. Actions Following the Identification of Intimate Partner Violence (IPV)	Immediate intervention protocols focused on safety and validation. This theme includes empathetic, nonjudgmental listening, activation of multidisciplinary referral pathways, respectful emotional support, and the creation of accessible safe spaces for those experiencing violence.

Source: Authors’ own elaboration.

**Table 3:** Analytical hierarchy: Trajectory from initial codes to themes and final conceptual framework.

Raw Data Sources	Initial Codes	Categories	Themes	Final Conceptual Framework
Focus Groups & Reflective Journals	Fear of disclosure Shame and guilt Romantic love myths Normalization of control Lack of awareness	Psychological barriers Sociocultural normalization of violence	Theme 1: Barriers to Identifying Dating Violence	Integrated Conceptual Framework: Early identification and management of dating violence requires a trauma-informed, person-centred, multidisciplinary, and digitally informed approach addressing psychological, sociocultural, technological, and organizational factors.
	Social media monitoring Digital surveillance Location tracking Lack of privacy Lack of training Professional insecurity Limited protocols Biomedical approach	Technology-facilitated forms of control	Theme 2: Challenges in Addressing Intimate Partner Violence (IPV)	
	Digital literacy Peer support Educational strategies	Professional and organizational barriers	Theme 3: Potential Strategies for Identifying Dating Violence	
	Validation of experiences Multidisciplinary collaboration Peer support Safe spaces	Facilitators for identification and intervention Prevention and educational strategies	Theme 4: Actions Following the Identification of Intimate Partner Violence (IPV)	

Source: Authors’ own elaboration.

## Data Availability

Due to ethical restrictions, the data supporting the findings of this study cannot be made publicly available.

## Supplementary Materials

The following supporting information can be downloaded at: https://www.mdpi.com/article/10.3390/nursrep16090319/s1, Three supplementary materials accompany this manuscript: Supplementary Material S1 (Thematic Guide for Focus Groups), Supplementary Material S2 (Reflective Diary Template), and Supplementary Material S3 (Consolidated Criteria for Reporting Qualitative Research [COREQ] Checklist).

## Public Involvement Statement

No public involvement in any aspect of this research.

## Guidelines and Standards Statement

This manuscript was drafted in accordance with the Consolidated Criteria for Reporting Qualitative Research (COREQ) [12] for qualitative research involving focus groups and reflective written data. (Supplementary File S3).

## Use of Artificial Intelligence

The authors acknowledge the use of the latest available version of Microsoft Copilot (sobre GPT-5) (accessed through the Microsoft Copilot interface) solely for assistance with figure conceptualization, language refinement, and graphical formatting. No artificial intelligence tool was used for data generation, data analysis, statistical processing, qualitative interpretation, or scientific decision-making. All AI-assisted materials were critically reviewed and verified by the authors, who remain fully responsible for the content, interpretation, and conclusions presented in this manuscript.
