# Supplementary material for: Perceived Healthcare Interventions and Clinical Strategies Against Dating Violence: A Qualitative Exploration of Expert and Nursing Perspectives
Source: Nurs Rep. 2026 Sep 4;16(9):319. doi: 10.3390/nursrep16090319 (PMC13610462; doi:10.3390/nursrep16090319)
Supplement: Supplementary file 1 [file nursrep-16-00319-s001.zip › nursrep-4475112-supplementary update/Supplementary Material S1 & S2.pdf]

S1. Thematic Guide for Focus Group

Description: This thematic guide was developed to support data collection in focus groups with experts in dating violence.

Instructions for moderator: The moderator should facilitate discussion in an open and flexible manner, allowing participants to elaborate on their experiences. Probing questions are suggested to deepen responses. Reflexive notes should be recorded after each session.

Block 1. Identification of types of violence in dating relationships

- Opening question: What types of violence do you identify in young couples?
- Experiences: Can you share professional experiences related to dating violence?
- Physical violence: How is physical violence manifested or detected?
- Psychological violence: What forms does psychological violence take?
- Digital violence (mobile/social media): What role does technology play?
- Control and harassment: What controlling behaviours are most common?
- Degradation and contempt: How are these forms expressed?
- Aesthetic pressure: Do you detect pressures related to body image or appearance?
- Probes: Can you give examples? How do victims describe these situations?

Block 2. Strategies and recommendations for addressing violence

- Opening question: What strategies are most effective in addressing dating violence?
- Detection guidelines: What indicators help identify cases early?
- Professional practices: How do you usually intervene?
- Co-creation: What key elements should be included in a prevention decalogue?
- Prevention recommendations: What messages are essential for young people?
- Communication strategies: How would you adapt this content into a podcast or digital format?
- Probes: What works in practice? What does not work?

Reflexivity

- How do your professional experiences influence your interpretation of dating violence?
- Did any discussion challenge your assumptions?
- How might the group dynamics have influenced the discussion?

S2. Reflective Diary Template

Description: This reflective diary template was designed to support reflexivity during qualitative data collection, in line with COREQ guidelines. It aims to capture professional perceptions, experiences, and decision-making processes when interacting with women affected by gender-based violence.

Instructions for participants: Participants are invited to complete this diary after each relevant interaction or at the end of the working day. Completion is flexible; only relevant sections need to be addressed.

General information (optional)

- Date
- Context of interaction (e.g., consultation, emergency, follow-up)
- Type of contact (face-to-face, telephone, other)

1. Description of the interaction

- Briefly describe the situation
- Main reason for the consultation or interaction
- Was gender-based violence identified or suspected?

2. Professional perceptions

- What elements led to suspicion or confirmation?
- Relevant indicators observed
- How would you describe the therapeutic relationship?

3. Intervention performed

- Actions taken
- Protocols applied (if any)
- Referral or follow-up conducted

4. Reflexivity

- What thoughts arose during the interaction?
- How might your professional background or assumptions have influenced the interaction?
- What emotions or internal reactions emerged?

5. Barriers and facilitators

- What challenges were encountered?
- What factors supported the intervention?

6. Learning and improvement

- What would you do differently in a similar situation?
- What competencies or resources are needed?

7. Additional comments

- Open field for any relevant reflections
