# Supplementary material for: Perceived Healthcare Interventions and Clinical Strategies Against Dating Violence: A Qualitative Exploration of Expert and Nursing Perspectives
Source: Nurs Rep. 2026 Sep 4;16(9):319. doi: 10.3390/nursrep16090319 (PMC13610462; doi:10.3390/nursrep16090319)
Supplement: Supplementary file 1 [file nursrep-16-00319-s001.zip › nursrep-4475112-supplementary update/Supplementary Material S3.pdf]

Suported Material S3

Table S1. Consolidated criteria for reporting qualitative studies (COREQ): 32-item checklist

**No**	**Item**	**Guide questions/description**	**Response**	**Checklist**
**Domain 1: Research team and reflexivity**<br>Personal Characteristics				
1.	Interviewer/ facilitator	Which author/ s conducted the interview or focus group? Invest	Focus groups were facilitated by the principal investigator (MPL).	Section 2.2
2.	Credentials	What were the researcher's credentials? E.g. PhD, MD	MPL and SSB held PhD degrees.	Section 2.2
3.	Occupation	What was their occupation at the time of the study?	University faculty members and researchers in nursing and IPV.	Section 2.2
4.	Gender	Was the researcher male or female?	Researchers were women.	Author informati on
5.	Experience and training	What experience or training did the researcher have Relationship with participants	MPL had expertise in egalitarian relationships and extensive experience conducting focus groups; SSB specialised in IPV research.	Sections 2.2, 2.3
Relationship with participants				
6.	Relationship established	Was a relationship established prior to study comm	Some researchers and participants already knew one another through their involvement in the University Equality Committee.	Section 2.2
7.	Participant knowledge of the Interviewer	What did the participants know about the researcher? eg interviewer research	Participants were informed about the study objectives and the researchers' role in the project.	Sections 2.1, 2.2
8.	Interviewer characteristics	What characteristics were reported about reasons and interests in the research topic	Researchers acknowledged that their backgrounds in egalitarian relationships, mental health, and gender-based violence could influence interpretation. Reflexive strategies were used to minimise bias.	Section 2.3
**Domain 2: study design**<br>Theoretical framework				

9.	Methodological orientation and Theory	What methodological orientation was stated to underpin the study? e.g. grounded theory, discourse analysis, ethnography, phenomenology, content analysis	Constructivist paradigm; interpretative phenomenological analysis (IPA) and inductive thematic analysis.	Sections 2, 2.2
**Participant selection**				
10.	Sampling	How were participants selected? convenience, consecutive, snowball	Convenience sampling based on professional relevance.	Section 2.1
11.	Method of approach	How were participants approached? eg. face-to face, telephone, mail,	Participants were contacted through healthcare institutions, community organisations, academic institutions, and professional networks.	Section 2.1
12.	Sample size	How many participants were in the study?	12 professionals.	Section 2.1
13.	Non-participation Setting	How many people refused to participate or dropped out? Reasons?	Two invited individuals did not complete informed consent and one could not attend due to clinical work commitments.	Section 2.1
**Setting**				
14.	Setting of data collection	Where was the data collected?	Private room at the Faculty of Nursing, University of Barcelona.	Section 2.2
15.	Presence of non-participants	Was anyone else presen besides the participants and researchers?	No non-participants were reported as being present.	Section 2.2
16.	Description of sample	What are the important characteristics of the sample?	Women aged 33–72 years from nursing, psychology, academia, community organisations, and healthcare services. Characteristics reported in Table 1.	Section 2.1; Table 1
**Data collection**				
17.	Interview guide	Were questions, prompts, guides provided by the authors? Was it pilot tested?	A thematic guide based on literature was used (Supplementary Material S2).	Section 2.2
18.	Repeat interviews	Were repeat interviews carried out? If yes, how many?	No repeat focus groups were conducted. Reflective journals were used as a complementary data source.	Section 2.2

19.	Audio/visual recording	Did the research use audio or visual recording to collect the data?	Focus groups were audio-recorded.	Section 2.2
20.	Field notes	Were field notes made during and/or after the interview or focus group?	Reflective field journals were maintained throughout the study.	Section 2.3
21.	Duration	What was the duration of the interviews or focus group?	90–120 minutes.	Section 2.2
22.	Data saturation	Was data saturation discussed?	Consensus-based saturation was reported during analysis.	Section 2.3
23.	Transcripts returned	Were transcripts returned to participants for comment and/or correction?	Transcripts were returned to participants for comment or correction	Section 2.3
**Domain 3: analysis and findings**<br>Data analysis				
24.	Number of data coders	How many data coders coded the data?	Coding and categorisation were conducted by the research team through consensus-based discussion, coordinated by the principal investigator.	Section 2.2
25.	Description of the coding tree	Did authors provide a description of the coding tree?	Categories and themes are presented in Table 2.	Results; Table 2
26.	Derivation of themes	Were themes identified in advance or derived from the data	Themes were derived inductively from the data within an IPA and thematic analysis framework.	Section 2.2
27.	Software	What software, if applicable, wns used to manage the data?	Atlas.ti®.	Section 2.2
28.	Participant checking	Did participants provide feedback on the findings	Member checking was conducted by presenting preliminary findings to participants.	Section 2.3
29.	Quotations presented	Were participant quotations presented to illustrate the themes/findings? Was each quotation identified? e.g., participant number.	Participant quotations were used throughout the Results section and identified by participant codes (e.g., P1–P14).	Results

30.	Data and findings consistent	**Was there consistency between the data presented and the findings?**	The findings are supported by quotations and are consistent with the reported themes.	Results
31.	Clarity of major themes	**Were major themes clearly presented in the findings?**	Four major themes are clearly presented and described.	Results; Table 2
32.	Clarity of minor themes	**Is there a description of diverse cases or discussion of minor themes?**	Subthemes and nuanced perspectives are presented within each major theme.	Results

Tong, A., Sainsbury, P., & Craig, J. (2007). Consolidated criteria for reporting qualitative research (COREQ): A 32-item checklist for interviews and focus groups. International Journal for Quality in Health Care, 19(6), 349–357. https://doi.org/10.1093/intqhc/mzm042
